# Packing Activated Carbons into Dense Graphene Network by Capillarity for High Volumetric Performance Supercapacitors

**DOI:** 10.1002/advs.201802355

**Published:** 2019-05-08

**Authors:** Pei Li, Huan Li, Daliang Han, Tongxin Shang, Yaqian Deng, Ying Tao, Wei Lv, Quan‐Hong Yang

**Affiliations:** ^1^ Nanoyang Group State Key Laboratory of Chemical Engineering School of Chemical Engineering and Technology Tianjin University Tianjin 300350 China; ^2^ Collaborative Innovation Center of Chemical Science and Engineering (Tianjin) Tianjin 300072 China; ^3^ Shenzhen Key Laboratory for Graphene‐based Materials Graduate School at Shenzhen Tsinghua University Shenzhen 518055 China

**Keywords:** activated carbons, capillary shrinkage, compact graphene network, supercapacitors, volumetric performance

## Abstract

Supercapacitors are increasingly in demand among energy storage devices. Due to their abundant porosity and low cost, activated carbons are the most promising electrode materials and have been commercialized in supercapacitors for many years. However, their low packing density leads to an unsatisfactory volumetric performance, which is a big obstacle for their practical use where a high volumetric energy density is necessary. Inspired by the dense structure of irregular pomegranate grains, a simple yet effective approach to pack activated carbons into a compact graphene network with graphene as the “peels” is reported here. The capillary shrinkage of the graphene network sharply reduces the voids between the activated carbon particles through the microcosmic rearrangement while retaining their inner porosity. As a result, the electrode density increases from 0.41 to 0.76 g cm^−3^. When used as additive‐free electrodes for supercapacitors in an ionic liquid electrolyte, this porous yet dense electrode delivers a volumetric capacitance of up to 138 F cm^−3^, achieving high gravimetric and volumetric energy densities of 101 Wh kg^−1^ and 77 Wh L^−1^, respectively. Such a graphene‐assisted densification strategy can be extended to the densification of other carbon or noncarbon particles for energy devices requiring a high volumetric performance.

Supercapacitors, which store energy by the adsorption of electrolyte ions on a porous electrode, have attracted widespread attention in the energy storage field. This is mainly due to their high power density and long life.^1^, ^2^, ^3^, ^4^, ^5^ In recent years, volumetric performance has become more important for energy storage in a limited space, because it is the prior concern for many applications, typical examples being portable electronics such as mobile phones, wearable electronics, biosensors, and nanorobotics.^6^, ^7^, ^8^, ^9^, ^10^, ^11^ Activated carbons (ACs),^12^, ^13^, ^14^ carbon nanotubes,^15^ graphene^16^, ^17^ or activated graphene,^18^ and other porous carbon materials^19^, ^20^ are potential candidates for supercapacitors mainly because of their large specific surface areas (SSAs) and acceptable electrical conductivity. Among the carbonaceous materials, ACs are the most commonly used owing to their abundant porosity and low cost, and thus have been already commercialized.^1^, ^5^, ^21^ However, a high volumetric energy density (*E*_v_) not only requires a high gravimetric capacitance (*C*_g_), but also a high electrode density and a wide operation voltage (*E*_v_ = ρ × *C*_g_ × (*∆U*)^2^/2).^9^ Recently, organic and ionic electrolytes were found to have a wide voltage window thanks to their high electrochemical stability.^22^ However, the electrode density still remains low due to the existence of macropores and interparticle voids in the porous carbons,^23^, ^24^ which is a big obstacle for high volumetric performance. Mechanical compression is a simple and normally used method to increase the density by eliminating interparticle voids, however, it may cause collapse of the internal pores and reduce the accessible surface area and the number of conducting paths hence leading to a decreased performance.^10^ So much effort has recently been made to increase the intrinsic densities of carbon materials with unimpeded ion transport channels by the dense assembly of graphene membranes, graphene monoliths, etc.^25^, ^26^, ^27^, ^28^, ^29^, ^30^, ^31^ However, the high cost and complex synthesis still restrict their practical uses. Therefore, the densification of low cost commercial ACs offers a practical way to improve the volumetric performance of supercapacitors.

Pomegranates own an ideally densely packed structure of irregular particles (**Figure** **1**a). In spite of inhomogeneous sizes and shapes, pomegranate grains are packed in a compact way without voids. As a result, the internal space of its rind is fully exploited to store as much flesh as possible. Inspired by pomegranate, it is expected that the irregular AC particles can be highly dense‐packed with minimal voids in a similar way. In this way, the more active materials are accommodated in a certain space, the higher energy is provided in a limited volume. To realize the dense packing like pomegranate particles, AC particles as “pomegranate grains” demand for some kind of “peel” for package and a compression force to facilitate the densification. To make the AC particles into a compact integration, a “peel” is highly desired to wrap them in a compact way, which is supposed to be 1) sheet‐like, 2) flexible, 3) strong, 4) electrically conductive, and 5) able to lap over each other as well.

**Figure 1:**
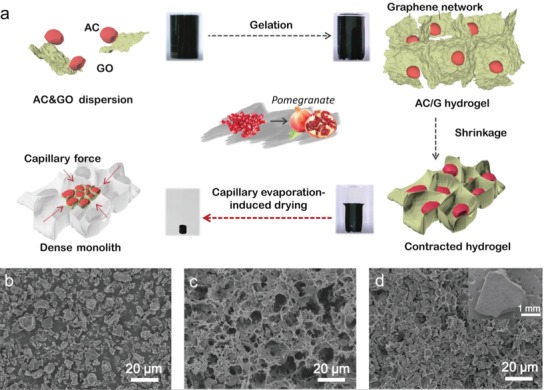
Pomegranate‐inspired densely packed structure for the densification of ACs. a) Scheme of how to pack activated carbons in a 3D graphene network. Inspired by the dense packing structure of pomegranate (middle), the YP80/G hydrogel was formed into a 3D structure through gelation, followed by its shrinkage in the solution as the reduction proceeded. The capillary evaporation‐induced drying (CEID) on such a hydrogel results in an obvious volume shrinkage to produce a dense monolith. SEM images of b) the low‐density YP80 powder and c) the low‐density YP80/G composite foam after freeze drying, and d) the high‐density YP80/G monolith after CEID.

Graphene would be an ideal candidate for such a peel due to its high strength and good conductivity,^3^ its flexibility to assemble into a 3D network^32^ and cling to the irregular particles, and more importantly, the shrinkage of the assembled graphene network caused by the capillary evaporation of water adsorbed inside it.^25^ As a proof of concept, we used commercial AC YP80 as a typical example to show the effectiveness of this densification strategy where a small amount of graphene is used and the self‐shrinkage of the graphene “peel” exerts a force to densify the ACs inside. As expected, this densification results in a compact structure with a relatively high electrode density of 0.76 g cm^−3^ that is much larger than the 0.41 g cm^−3^ for pristine ACs. Besides, the AC and G work synergistically for a higher capacitance, with the former providing a porous structure for ions storage and avoiding G layer restacking, and the latter constructing a 3D conductive network and modifying the ion transport. When used as an additive‐free supercapacitor electrode in an ionic liquid electrolyte, the graphene‐wrapped ACs show a gravimetric capacitance of 181 F g^−1^ and a volumetric capacitance of up to 138 F cm^−3^, resulting in high gravimetric and volumetric energy densities of 101 Wh kg^−1^ and 77 Wh L^−1^, respectively.

Figure 1a shows the AC densification strategy using the graphene “peel.” Typically, irregular AC particles were dispersed with amphiphilic graphene oxide (GO) sheets and then mixed with reductant L‐ascorbic acid sodium salt (L‐AASS) in a mixed solvent of water and ethanol. As the GO gradually reduced during the gelation process, the sheets overlapped to form an interconnected 3D network that wrapped AC particles in it. After shrinkage to form a hydrogel, it was densified by capillary evaporation‐induced drying (CEID) to produce a compact AC/G composite. The densification strategy composed of three main steps shown in Figure 1a has following key points: 1) The self‐assembly of G was clearly separated into two stages: gelation and shrinkage, which means that 3D network was firstly constructed before shrinkage. 2) The gelation time determines the graphene coating efficiency of AC, and we can make it up to 100% here even if a pretty small amount of graphene is used. The shrinkage associates with the reduction degree of GO and greatly influences the strength of hydrogel. 3) Compared to freeze drying which produces a foam‐like structure that retains the volume of hydrogel, we earlier developed a CEID approach that densifies the graphene network by the evaporation of water exerting a strong inward force on the graphene nanosheets.^25^ As the water evaporates from inside the network, the graphene composite gel shrinks drastically, making the monolith compact and mechanically strong. Taking YP80 as a typical example (Figure 1b), by analyzing factors of the three steps, we proposed pointers on how to control a scalable preparation process (Figure S1, Supporting Information) for graphene‐coated/encapsulated materials realized with 3D hydrogel, not confined to densification of AC.

The gelation process determines if all the particles can be packed in the graphene network. In our previous work, a pure graphene hydrogel was prepared by hydrothermal reduction of graphene oxide (GO) suspension (180 °C in water in a sealed autoclave) and then treated by CEID. However, the hydrothermal process was found to be ineffective here when hydrophobic AC particles were intended to be packed into graphene network through gelation (Figure S2 and Table S1, Supporting Information). We have therefore developed a chemical‐reduction‐induced assembly method for the G‐assisted gelation of an AC suspension, in which ACs and GO were dispersed in a water/ethanol mixture and gelation occurs at a low temperature (normally at 80 °C) in ambient atmosphere. This unique gelation process is critical for the later capillary shrinkage of the resulting gel. When this low‐temperature reduction was used to prepare a composite gel, the reduced GO (rGO) sheets became less hydrophilic and tend to overlap and form a 3D network^33^ with AC particles evenly distributed among the sheets. A cylindrical gel was observed at the very beginning of shrinkage (Figure 1a and Movie S1, Supporting Information), so it can be inferred that the gelation was completed ahead of shrinkage and explain why hydrogel shapes are always consistent with the shape of containers (Figure S3, Supporting Information). In this period, which took 2.5 h in our experiment with YP80, there was no obvious external change visible to the naked eye (Movie S1, Supporting Information) though a 3D network has been constructed and a stagnant gel‐like state was formed. Once the particles were supported by the network, they would no longer sink to the bottom.

Note that not all AC particles could be packed if gelation took so long that ACs will be partially precipitated. That is the reason why the hydrothermal reduction was not efficient here and thus made it difficult to control composition of the composites. Although it was reported that a G hydrogel could form within an hour using the hydrothermal method,^33^ but more time is needed when a different solvent is used. In order to disperse the AC particles, a mixture of deionized water and ethanol was chosen. Gelation time of GO (1 mg mL^−1^) in the ethanol/water mixture (1.5 h, Movie S2, Supporting Information) is twice that in water (40 min, Movie S3, Supporting Information), due to the increased affinity of the mixed solvent. After AC was added, the gelation time increased even more (2.5 h, Movie S1, Supporting Information) because the AC particles between the graphene sheets increased the layer separation and hindered the gelation. Except for the solvent and packed materials, other factors involving the reduction of GO, like initial oxygen content, temperature, and reductant, and interaction between rGO sheets, such as concentration, are also important to adjust the gelation time (Figure S4, Supporting Information). In this work, the GO concentration of 1 mg mL^−1^ with an L‐AASS reductant concentration of 0.01 m at 80 °C for 16 h were chosen as the optimized condition to prepare the YP80/G composite.

Shrinkage occurred in the solution as the number of oxygen‐containing functional groups further decreased, causing stronger π−π interaction between the sheets and a continuous shrinkage of the network. The gel became smaller during the following 5 h shrinkage process but then remained its size because reduction had ended (Movie S1, Supporting Information). It was found that the solvent had a great influence on the shrinkage speed (Movies S2 and S3, Supporting Information). Shrinkage in the pure water was quite fast (50 min) whereas in the mixed solvent it took 5 h. The shrinkage speed is greatly influenced by the interaction between the rGO sheets and the solvent.^34^ In the pure water, the rGO sheets have lower binding energy with the water molecules, which tend to be isolated from the water suspension. This favors the π–π interactions between sheets and hence results in a fast shrinkage. However, in the water/ethanol solvent, a stronger interaction between rGO and ethanol molecules actually restrains the crosslinking of rGO layers.

CEID played a vital role in the dense packing of ACs, since there was still a lot of empty space in the network after shrinkage in the solution, as shown in the scanning electron microscope (SEM) images of freeze‐dried samples (Figure 1c). During CEID, solvent molecules were removed and the graphene network contracted as a result of capillary forces^25^ and this forced the AC particles into a compact arrangement. Every “pocket” in the G network in which one or more AC particles settled shrank so that the flexible G clung to the surface of the irregular AC particles (Figure 1d and Figure S5a,b, Supporting Information). As a whole, the network formed a strong monolith with the smallest number of voids. Unlike the loose foam structure produced by freeze drying (Figure S5c, Supporting Information), this compact monolith had a smooth texture and appearance (low‐magnification SEM images shown in Figure 1d). Despite the marked differences in apparent density, the samples dried by the two entirely different methods have similar SSAs and pore distributions (Figure S6a,b, Supporting Information), while XRD of the freeze‐dried sample has a broader peak at around 24.4° (Figure S6c, Supporting Information), indicating a more disordered structure.

As shown in **Figure** **2**a, the composite materials were prepared by adjusting the mass ratio of YP80 and GO (details are listed in Table S1 of the Supporting Information). A cylindrical gel was formed even if a pretty small amount of G was used to package the YP80 particles, yielding the sample with 96.7% YP80 in the final material. However, without sufficient G sheets to form a large network, the gel of this last sample had a deformed cylindrical shape with a thick bottom owing to particle deposition. As the YP80 content increases, N_2_ adsorption–desorption isotherms of the samples begin to show more YP80 features as shown in Figure 2b, with a hysteresis loop from G gradually disappearing while a plateau getting higher thanks to an increase in micropores. Note that the G sheets, whose diameters are hundreds of nanometers, have a great influence on the way the YP80 particles are stacked, but do not alter their pore structures (Figure 2c and Table S2, Supporting Information), so the calculated BET SSAs based on the mass ratios are close to the experimental values (Figure 2d). From the pore size distribution in Figure 2c, it is also found that there are a large number of micropores around 1 nm and small mesopores around 2 nm in the YP80/G, which are suitable for the storage of EMIMBF_4_ ions.^35^, ^36^ Notably, there is a definite dependence of an electrode conductivity on the proportion of G, since G has a much better conductivity than YP80 (Figure 2e). Taking the 76.5% sample as an example, its conductivity is six times that of pristine YP80.

**Figure 2:**
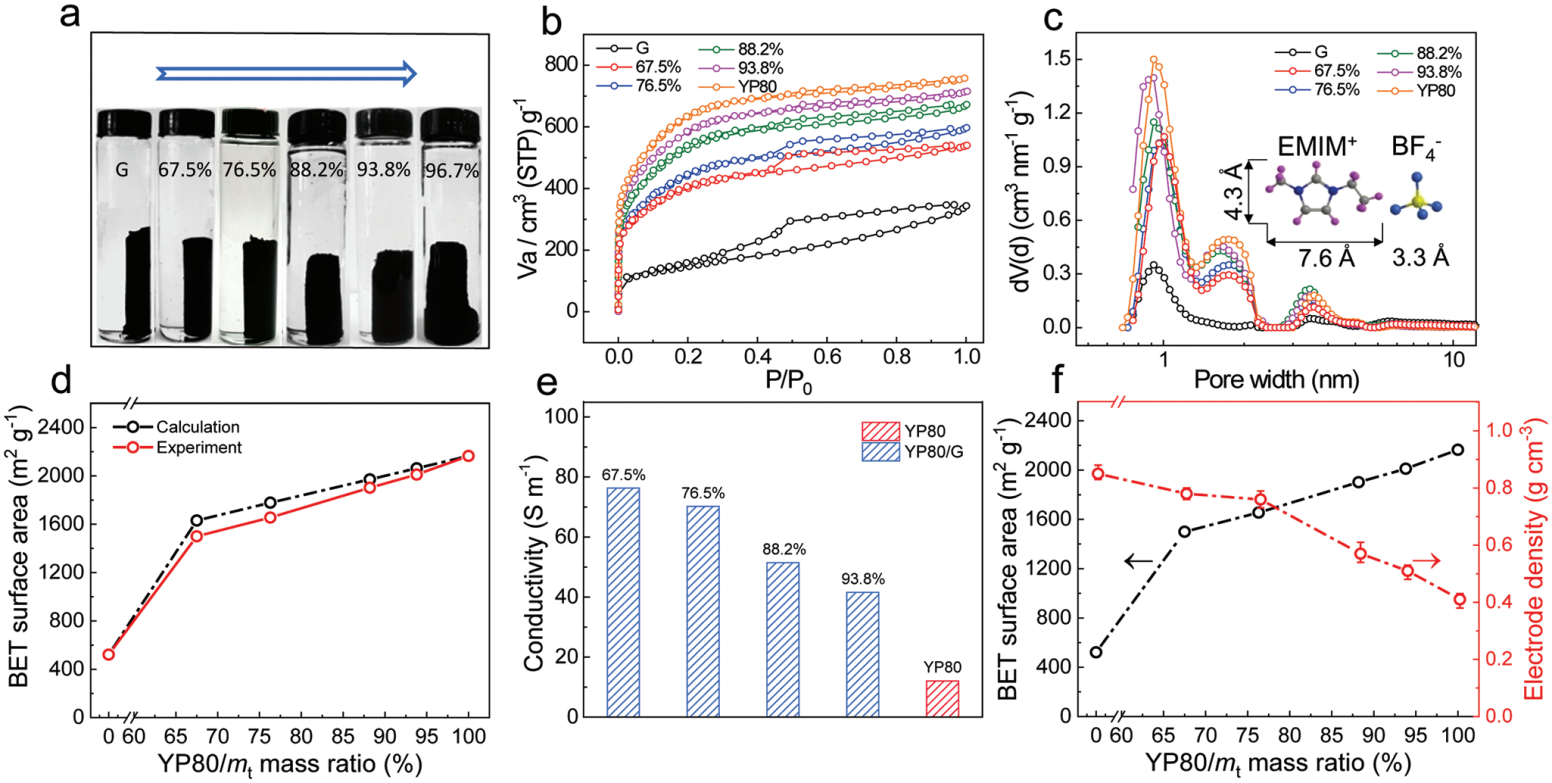
Porosity and density of YP80/G with different mass ratios. a) Photographs of YP80/G hydrogels before capillary shrinkage. This assembly method is effective even using a small amount of graphene. However, a regular gel cannot be obtained once the proportion of G decreases dramatically. The blue arrow indicates an increase of the YP80 mass fraction in the product. b) N_2_ adsorption–desorption isotherms. c) Pore size distributions (DFT), inset: models of the structure of EMIM^+^ and BF_4_^−^ ions. d) Experimental and calculated BET SSAs where the calculations are based on the mass ratios. e) Conductivity of electrodes (including binder) measured by the four‐point probe method. f) Balance between SSA and density. Higher YP80/*m*_t_ mass ratios lead to a higher surface area and a lower density.

Figure 2f shows that an increase in the wt% of YP80 leads to an increase of SSA from 521 to 2165 m^2^ g^−1^, with a decrease in electrode density from 0.85 to 0.41 g cm^−3^. Obviously, a higher proportion of micropore‐rich YP80 produces a higher SSA. However, a certain amount of G is needed to form a well‐connected network to pack and compress AC particles to obtain a dense bulk material (Figure S7a,b, Supporting Information). If there is insufficient G, there are breaks in the G network (Figure S7c, Supporting Information) and this has a detrimental effect on the strength of the compression exerted by the graphene sheets to rearrange the AC particles, leading to a lower density. It is thus important to balance the SSA and density. Figure 2f shows that the SSA increases sharply as the AC content reaches 76.5 wt%. A higher AC content results in a moderate increase in surface area but a significant decrease in density. Therefore, 76.5% YP80/G is the optimized sample that can well balance SSA and electrode density, and it exhibits the highest volumetric capacitances of the composite electrodes with different YP80 mass loadings as expected (Figure S8, Supporting Information). At this point, the composite has a SSA of 1655 m^2^ g^−1^ and an electrode density of 0.76 g cm^−3^. The following parts for the discussion of YP80/G are all based on 76.5% YP80/G.

The monolithic form was maintained after the cylindrical wet gel experienced CEID at room temperature and annealing at 800 °C for an hour (**Figure** **3**a). The bulk material has a remarkable mechanical strength, for a 76.5% YP80/G monolith with a diameter of around 0.85 cm can support 500 g. This confirms that graphene sheets that have excellent stiffness are able to overlap into a network with an outstanding mechanical performance. The tapping density of the material is calculated to be 0.55 g cm^−3^, which is twice that of YP80 (0.27 g cm^−3^). The SEM image in Figure 3a shows the compact packing structure of YP80 and close connection between the YP80 particles and the graphene sheets. In this composite, in addition to the primary role of packing the AC particles, the 3D graphene network provides conductive paths through the composite, and the sheets clinging to the surface of the AC particles. This face‐to‐face conductive contact effectively reduces the particle contact resistance, which is a major contributor to the resistance of aggregated carbon powders.^37^, ^38^ In addition, the AC particles between the sheets prevent the graphene layers from restacking during electrode manufacture and electrochemical cycling, thus making more effective surface area available for charge storage. Inevitably, there are still some small cracks between the graphene sheets and the AC particles as seen in the SEM, but as the spaces in this dense composite structure, it will actually facilitate ion transport as an ion channel. As an amorphous carbon, YP80 cannot form π–π interactions with graphene sheets and it only has small amounts of oxygen‐containing functional group on its surface (3.8% as verified by TGA curve in Figure S9 and the XPS results in Table S3 of the Supporting Information). It is hard for YP80 to form covalent or hydrogen bonds with graphene sheets. Hence, there should be a physical combination between AC and graphene sheets, rather than chemical bonding.

**Figure 3:**
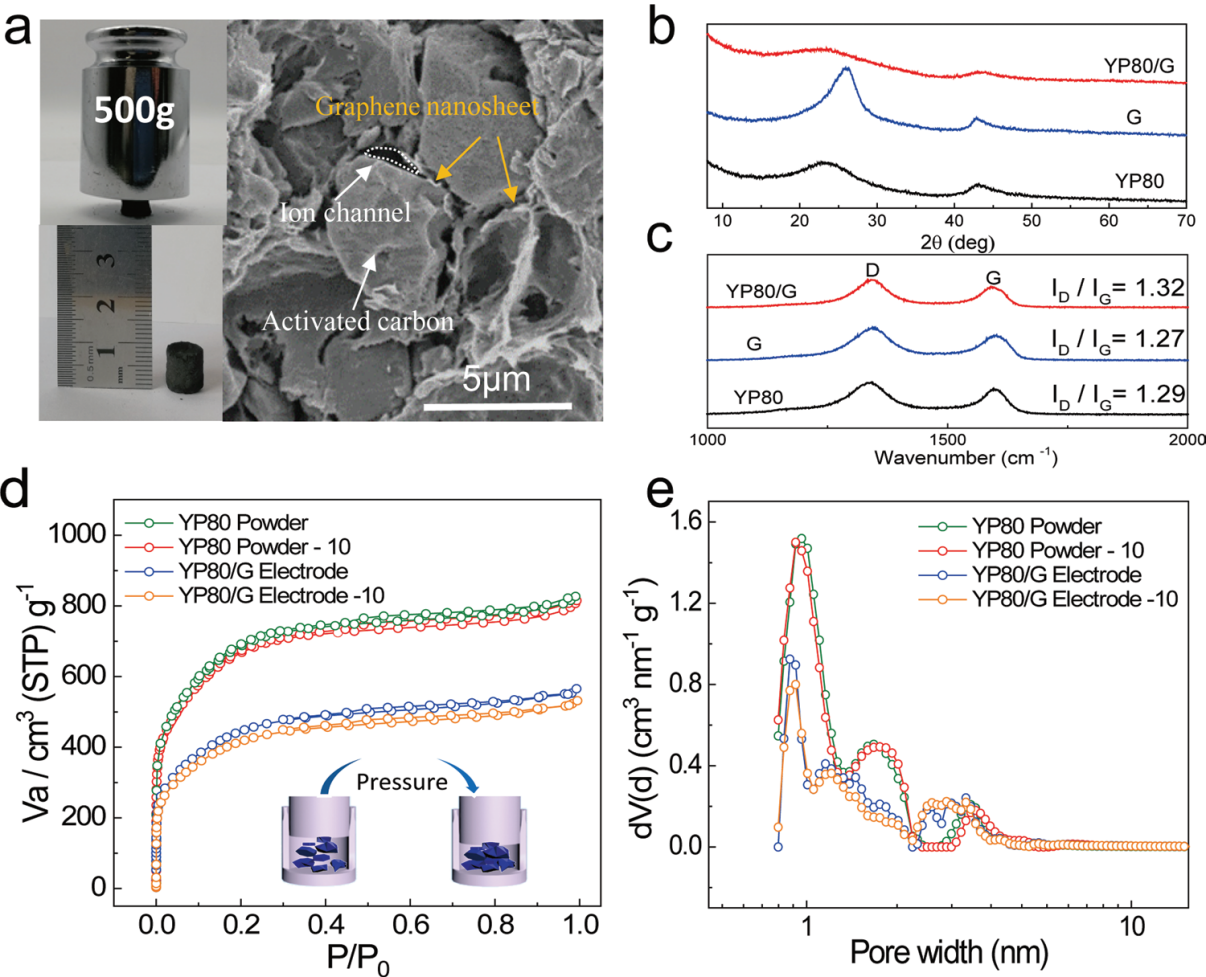
The structure and properties of optimized YP80/G. a) Photograph of a dense graphene building block (made of 300 mL mixed solution) supporting weight and SEM image of the composite. b) XRD patterns of YP80, G, and YP80/G. c) Raman spectra of YP80/G, YP80, and G. d) N_2_ adsorption–desorption isotherms. e) Pore size distributions (DFT) of uncompressed and compressed YP80 power and YP80/G electrodes, inset: schematic of the compressing process. The pressure used for connecting the electrode and current collector will not alter the porosity of the composite structure.

From the X‐ray diffraction (XRD) patterns in Figure 3b, a broad peak for YP80 at around 24.4° (002) indicates its amorphous nature and low degree of graphitization. In contrast, the more intense (002) peak in the G sample indicates higher graphitic ordering with an interlayer spacing of 3.45 Å. This peak is weaker in the composite because the stacking of graphene sheets is prevented by the presence of the YP80 particles. In Raman spectroscopy (Figure 3c), since YP80 is a microcrystalline carbon, an obvious G band, which represents *sp* ^2^‐hybridized carbon atoms, is present, while a stronger D band, attributed to disorder, indicates its long‐range disordered structure, consistent with the XRD results. After compositing the two carbon materials the *I*_D_/*I*_G_ value is slightly higher because the disorder degree of graphene increases due to the insertion of YP80 between sheets. In addition, since it is important to determine whether compressing the electrodes causes the collapse of pores,^10^ N_2_ adsorption–desorption isotherms were measured before and after the YP80 powder and YP80/G electrode were compressed (Figure 3d). Here, YP80/G electrode refers to the calendared electrode containing active materials (YP80/G) and additives after mechanical compression (10 MPa) onto the current collector. Similar to uncompressed samples (2165 and 1492 m^2^ g^−1^), the specific surface areas of the YP80 powder and YP80/G electrode are 2147 and 1435 m^2^ g^−1^ after compressing, respectively (Table S4, Supporting Information). And they exhibit almost the same pore size distribution as shown in Figure 3e. Different from flexible graphene, whose pores are either collapsed or compressed to a smaller size by this treatment, thus causing a significant increase in density,^31^ the composite carbon retains its main porosity with a stable pore structure and surface area so its density increase caused by the pressure is greatly limited.

As shown in **Figure** **4**, the electrochemical performances were performed in a symmetric two‐electrode system in the ionic liquid of 1‐ethyl‐3‐methylimidazolium tetrafluoroborate (EMIMBF_4_). For comparison, the YP80 and graphene mixed powder (YP80/G‐P) with 76.5% YP80 was prepared without the above gelation process. As expected, the powder sample shows a low density of 0.51 g cm^−3^ due to the low density of graphene and YP80. In contrast, with the gelation process, YP80/G shows an obviously increased density of 0.76 g cm^−3^ (inset figure in Figure 4a). The thick restacked G layers in YP80/G‐P led to a lower SSA (1497 cm^2^ g^−1^) than YP80/G. Besides, the thick restacked G layers in YP80/G‐P is hard to be an efficient conductive additive due to their uneven distribution among the particles (Figure S10, Supporting Information), which is also unfavorable for the ion transport. Additive‐free electrodes of YP80/G were prepared thanks to the excellent conductive networks formed by the graphene sheets. The specific capacitance of YP80/G, calculated from galvanostatic charge/ discharge curves of cells charged at 4 V (Figure S11, Supporting Information), reaches 181 F g^−1^ at 0.2 A g^−1^, nearly comparable to YP80 (containing 10% conductive additive) and higher than that of YP80/G‐P, 152 F g^−1^ (Figure 4a). As the current density increases from 0.2 to 10 A g^−1^, the YP80/G exhibits an excellent rate performance and retains 56.1% of its initial capacitance at 10 A g^−1^ (102 F g^−1^), much higher than that of YP80 (37.8%) and YP80/G‐P (35.5%). Nyquist plots of the three samples (Figure 4b) show that they all have a more ideal capacitive behaviors because of the vertical curves in the low‐frequency region. According to the equivalent circuits, the YP80/G exhibits the lowest ohmic resistance (1.58 Ω) and charge transfer resistance (1.81 Ω) (Figure S12, Supporting Information). By contrast, the resistance in an ionic liquid electrolyte is higher than that in an organic electrolyte (Figure S13, Supporting Information) due to a higher viscosity, lower conductivity and larger ion size of ionic liquid, and YP80/G shows a high specific capacitance of 138 F g^−1^ at 10 A g^−1^ in 1 m EMIMBF_4_/AN. However, the neat ionic liquid electrolyte has the advantage of high‐voltage stability, resulting in a higher energy density.^21^, ^22^

**Figure 4:**
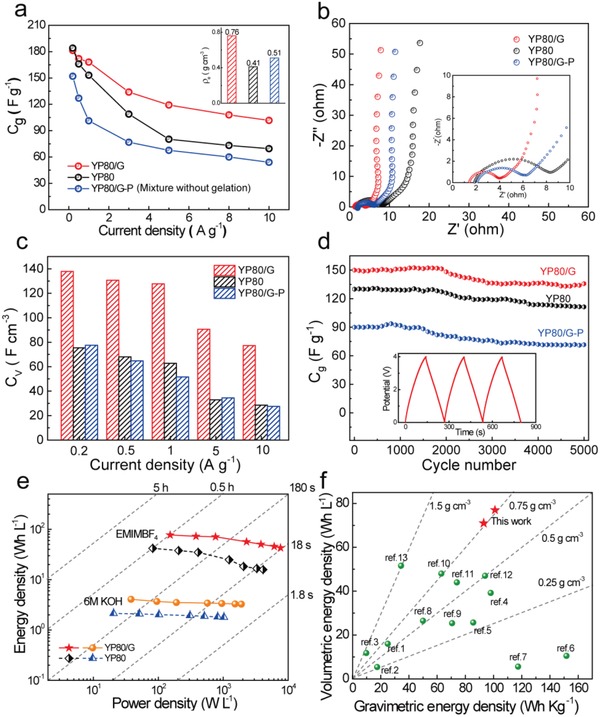
Electrochemical performance of the optimized YP80/G electrode in EMIMBF_4_. The YP80 and YP80/G‐P samples are shown for comparison. a) Gravimetric capacitances of the three samples at the current densities from 0.2 to 10 A g^−1^, inset: the electrode densities of the three samples. b) Nyquist plots, inset: the close‐up view of the high‐frequency regime. c) Volumetric capacitances versus different current densities. d) Cycling stability at a current density of 2 A g^−1^, the inset shows charge–discharge curves of YP80/G. e) Volumetric Ragone plots. f) Cartesian diagram of the volumetric energy density versus the gravimetric energy density, the two red stars are the energy densities at 0.2 A g^−1^ and 1 A g^−1^, respectively, in this work.

Bulk YP80/G material has an obvious advantage regarding to volumetric capacitances benefitting from excellent gravimetric capacitance and high electrode density among all samples. As shown in Figure 4c and Figure S14 (Supporting Information), YP80/G has the highest volumetric capacitance of 138 F cm^−3^ at 0.2 A g^−1^, much higher than that of YP80 (78 F cm^−3^), YP80/G‐P (75 F cm^−3^), YP80/G foam (60 F cm^−3^), and G (75 F cm^−3^). Such volumetric capacitance advantage is also demonstrated in organic and aqueous electrolytes (Figures S15 and S16, Supporting Information). The Figure 4d exhibits the cyclic stability of YP80/G, YP80/G‐P, and YP80. YP80/G retains 91% of its initial capacitance and keeps a stable surface morphology (Figure S17, Supporting Information) after 5000 cycles at a current density of 2 A g^−1^.

Since the porous AC forms the majority of the composite structure, its high SSA ensures a high gravimetric performance. At the same time, the more efficient packing of the AC particles produced by the graphene sheets and capillarity forces, maximizes the volumetric performance of the composite. The Ragone plots in Figure 4e shows that supercapacitors with YP80/G electrodes, which have an excellent gravimetric energy density of up to 101 Wh kg^−1^ (Figure S18a, Supporting Information), contribute to an increase in maximum volumetric energy density from 42 to 77 Wh L^−1^ compared to YP80 electrodes. Due to a superior balance of porosity and density, the YP80/G composite shows the highest volumetric energy density and power density among the samples (Figure S18, Supporting Information). Besides, the tight contact between graphene sheets and ACs particles enables an excellent conductive network and the high‐efficiency ion transport channels, which are two key factors in helping the compact supercapacitors maintain their rate performance. For further comparison, the YP80/G has a volumetric energy density of 43 Wh L^−1^ at a power of 7.6 kW L^−1^, while the YP80 exhibits 15 Wh L^−1^ at a power of 4.1 kW L^−1^. And the gravimetric and volumetric values both outperform carbon‐based materials existing in previously reported electrode materials (Figure 4f and Table S5, Supporting Information). Moreover, the dense structure ensures that there is sufficient but not redundant space for the electrolyte inside the electrode materials, which greatly promotes the utilization of the electrolyte and reduces the weight and volume fraction of the non‐electrochemically active components, thus improving the gravimetric and volumetric energy densities of the final device.^6^ Moreover, such a densification strategy can be also extended to other porous yet low‐density activated carbons (Figure S19, Supporting Information), which has great potential in terms of a high volumetric energy density.

We propose a general densification strategy for packing AC particles in a dense graphene monolith, inspired by the dense structure of pomegranate where irregular grains are compactly packed in the peel. The flexible graphene network package compressed by capillary forces achieve a compact arrangement of the particles and make the AC powder materials into dense bulk materials, while the porous texture of the ACs is well retained. By balancing the porosity and density of the AC/G, the optimized composite increases the electrode density to 1.9 times that of pure AC and achieves a high volumetric energy density of 77 Wh L^−1^. This densification strategy has great potential for use with other porous yet low‐density granular carbon materials besides ACs for the production of high volumetric energy storage devices, not limited to supercapacitors. Furthermore, to our best knowledge, as the first densification strategy reported for commercial ACs, this method is simple and easily scalable, promising to accelerate the development of industrial supercapacitors with higher volumetric energy densities.

## Conflict of Interest

The authors declare no conflict of interest.

## Supporting information

Supplementary

Supplementary

Supplementary

Supplementary

## References

[1] P. Simon , Y. Gogotsi , Nat. Mater. 2008, 7, 845. 18956000 10.1038/nmat2297

[2] P. Simon , Y. Gogotsi , B. Dunn , Science 2014, 343, 1210. 24626920 10.1126/science.1249625

[3] R. Raccichini , A. Varzi , S. Passerini , B. Scrosati , Nat. Mater. 2015, 14, 271. 25532074 10.1038/nmat4170

[4] M. R. Lukatskaya , B. Dunn , Y. Gogotsi , Nat. Commun. 2016, 7, 12647. 27600869 10.1038/ncomms12647 PMC5023960

[5] F. Wang , X. Wu , X. Yuan , Z. Liu , Y. Zhang , L. Fu , Y. Zhu , Q. Zhou , Y. Wu , W. Huang , Chem. Soc. Rev. 2017, 46, 6816. 28868557 10.1039/c7cs00205j

[6] Y. Gogotsi , P. Simon , Science 2011, 334, 917. 22096182 10.1126/science.1213003

[7] C. Zhang , W. Lv , Y. Tao , Q.‐H. Yang , Energy Environ. Sci. 2015, 8, 1390.].

[8] M. Beidaghi , Y. Gogotsi , Energy Environ. Sci. 2014, 7, 867.

[9] H. Li , Y. Tao , X. Zheng , J. Luo , F. Kang , H.‐M. Cheng , Q.‐H. Yang , Energy Environ. Sci. 2016, 9, 3135.

[10] C. Liu , X. Yan , F. Hu , G. Gao , G. Wu , X. Yang , Adv. Mater. 2018, 30, 1705713. 10.1002/adma.201705713 29537115

[11] X. Yang , C. Cheng , Y. Wang , L. Qiu , D. Li , Science 2013, 341, 534. 23908233 10.1126/science.1239089

[12] E. Frackowiak , F. Beguin , Carbon 2001, 39, 937.

[13] M. Sevilla , R. Mokaya , Energy Environ. Sci. 2014, 7, 1250.

[14] L. Yu , L. Hu , B. Anasori , Y.‐T. Liu , Q. Zhu , P. Zhang , Y. Gogotsi , B. Xu , ACS Energy Lett. 2018, 3, 1597.

[15] H. Pan , J. Li , Y. Feng , Nanoscale Res. Lett. 2010, 5, 654. 20672061 10.1007/s11671-009-9508-2 PMC2894167

[16] W. Lv , Z. Li , Y. Deng , Q.‐H. Yang , F. Kang , Energy Storage Mater. 2016, 2, 107.

[17] M. D. Stoller , S. Park , Y. Zhu , J. An , R. S. Ruoff , Nano Lett. 2008, 8, 3498. 18788793 10.1021/nl802558y

[18] Y. Zhu , S. Murali , M. D. Stoller , K. Ganesh , W. Cai , P. J. Ferreira , A. Pirkle , R. M. Wallace , K. A. Cychosz , M. Thommes , Science 2011, 332, 1537. 21566159 10.1126/science.1200770

[19] L. L. Zhang , X. S. Zhao , Chem. Soc. Rev. 2009, 38, 2520. 19690733 10.1039/b813846j

[20] T. Lin , I.‐W. Chen , F. Liu , C. Yang , H. Bi , F. Xu , F. Huang , Science 2015, 350, 1508. 26680194 10.1126/science.aab3798

[21] F. Béguin , V. Presser , A. Balducci , E. Frackowiak , Adv. Mater. 2014, 26, 2219. 24497347 10.1002/adma.201304137

[22] P. J. Hall , M. Mirzaeian , S. I. Fletcher , F. B. Sillars , A. J. Rennie , G. O. Shitta‐Bey , G. Wilson , A. Cruden , R. Carter , Energy Environ. Sci. 2010, 3, 1238.

[23] F. Xu , Z. Tang , S. Huang , L. Chen , Y. Liang , W. Mai , H. Zhong , R. Fu , D. Wu , Nat. Commun. 2015, 6, 7221. 26072734 10.1038/ncomms8221 PMC4490369

[24] M. F. El‐Kady , V. Strong , S. Dubin , R. B. Kaner , Science 2012, 335, 1326. 22422977 10.1126/science.1216744

[25] Y. Tao , X. Xie , W. Lv , D.‐M. Tang , D. Kong , Z. Huang , H. Nishihara , T. Ishii , B. Li , D. Golberg , Sci. Rep. 2013, 3, 2975. 24131954 10.1038/srep02975 PMC3797987

[26] S. Wu , Y. Zhu , Sci. China Mater. 2017, 60, 25.

[27] Y. Yoon , K. Lee , S. Kwon , S. Seo , H. Yoo , S. Kim , Y. Shin , Y. Park , D. Kim , J.‐Y. Choi , ACS Nano 2014, 8, 4580. 24680354 10.1021/nn500150j

[28] Y. Bu , T. Sun , Y. Cai , L. Du , O. Zhuo , L. Yang , Q. Wu , X. Wang , Z. Hu , Adv. Mater. 2017, 29, 1700470. 10.1002/adma.201700470 28417596

[29] D. N. Futaba , K. Hata , T. Yamada , T. Hiraoka , Y. Hayamizu , Y. Kakudate , O. Tanaike , H. Hatori , M. Yumura , S. Iijima , Nat. Mater. 2006, 5, 987. 17128258 10.1038/nmat1782

[30] Y. Zhou , M. Ghaffari , M. Lin , E. M. Parsons , Y. Liu , B. L. Wardle , Q. Zhang , Electrochim. Acta 2013, 111, 608.

[31] S. Murali , N. Quarles , L. L. Zhang , J. R. Potts , Z. Tan , Y. Lu , Y. Zhu , R. S. Ruoff , Nano Energy 2013, 2, 764.

[32] W. Lv , C. Zhang , Z. Li , Q. H. Yang , J. Phys. Chem. Lett. 2015, 6, 658. 26262482 10.1021/jz502655m

[33] Y. Xu , K. Sheng , C. Li , G. Shi , ACS Nano 2010, 4, 4324. 20590149 10.1021/nn101187z

[34] C. Luo , W. Lv , C. Qi , L. Zhong , Z. Z. Pan , J. Li , F. Kang , Q. H. Yang , Adv. Mater. 2018, 1805075. 10.1002/adma.201805075 30592336

[35] J. Chmiola , G. Yushin , Y. Gogotsi , C. Portet , P. Simon , P.‐L. Taberna , Science 2006, 313, 1760. 16917025 10.1126/science.1132195

[36] C. Largeot , C. Portet , J. Chmiola , P.‐L. Taberna , Y. Gogotsi , P. Simon , J. Am. Chem. Soc. 2008, 130, 2730. 18257568 10.1021/ja7106178

[37] A. Pandolfo , A. Hollenkamp , J. Power Sources 2006, 157, 11.

[38] A. Pandolfo , G. Wilson , T. Huynh , A. Hollenkamp , Fuel Cells 2010, 10, 856.

