# Supplementary material for: Packing Activated Carbons into Dense Graphene Network by Capillarity for High Volumetric Performance Supercapacitors
Source: Adv Sci (Weinh). 2019 May 8;6(14):1802355. doi: 10.1002/advs.201802355 (PMC6661934; doi:10.1002/advs.201802355)
Supplement: Supplementary file 1 — Supplementary [file ADVS-6-1802355-s002.pdf]

Copyright WILEY-VCH Verlag GmbH & Co. KGaA, 69469 Weinheim, Germany, 2019.

ADVANCED SCIENCE
Open Access

Supporting Information

for *Adv. Sci.,* DOI: 10.1002/advs.201802355

Packing Activated Carbons into Dense Graphene Network by Capillarity for High Volumetric Performance Supercapacitors

Pei Li, Huan Li, Daliang Han, Tongxin Shang, Yaqian Deng, Ying Tao, Wei Lv, and Quan-Hong Yang**

Supporting Information

Packing activated carbons into dense graphene network by capillarity for high volumetric performance supercapacitors

Pei Li‡, Huan Li‡, Daliang Han‡, Tongxin Shang, Yaqian Deng, Ying Tao, Wei Lv and Quan-Hong Yang**

I. Experimental sections

Preparation of YP80/G. Graphite oxide (GO) was prepared from graphite powder using a modified Hummers method, and the activated carbon was purchased from Kuraray Chemical in Japan (YP80, tap density: ~ 0.27 g cm^{-3}, BET specific surface area: 2160 m^2 g^{-1}). Typically, the GO and YP80 were separately dispersed in deionized water and alcohol, and after sonicating for 2h, the two kinds of dispersion were mixed in the same volume and sonicated for 15 mins. Then L-ascorbic acid sodium salt (L-AASS) was added before the sample was treated at 80℃ for 16h, resulting in a black cylindrical composite hydrogel. Next, the gel was immersed in deionized water for 24h to remove the salt in it, followed by being dried in a fume cupboard at room temperature. Finally, it was heated in a tubular furnace under a constant flow of hydrogen and argon at 800 °C to get the product YP80/G. In this work, seven samples were prepared and the raw material ratios of YP80 to GO were as follows: 0:10, 6:4, 5:5, 7:3, 8:2, 9:1, 10:0, which was controlled to achieve different amounts of YP80 within the YP80/G composite. The samples are named by the mass fraction of YP80 in the product.

Preparation of YP80/G-P, YP80 and G. For reference, a sample was prepared by the same process as YP80/G except that it was under stirring at 80 ℃ for 16h and thus no gel was formed. After the suspension was filtrated and washed by deionized water several times, it was dried and annealed at the same condition to get YP80/G powder (YP80/G-P). YP80 sample was heated under the same atmosphere at 800 °C before use. G was produced in a similar method as YP80/G but without YP80.

Characterization of materials. SEM images were captured on Hitachi S-4800 (Hitachi, Japan). Nitrogen adsorption measurement was conducted at 77 K using a BEL-mini instrument and a BEL-max instrument (BEL Inc., Japan). Pore volume was calculated at a relative pressure

P/P_0=0.990, the specific surface area was obtained by Brunauer-Emmett-Teller (BET) analyses of the adsorption isotherm and the pore size distribution was calculated using density functional theory (DFT). The electrical conductivities were measured by a standard four-point-probe resistivity measurement system (FT-341, Ningbo, China). XRD patterns were recorded on a Bruker D-8 (Cu Kα radiation, λ= 1.54056 Å) at room temperature and the data were collected from 5° to 70° with a scan rate of 10° min^{-1}. Raman spectra were recorded using a micro-Raman spectroscope (JY HR800) with 532.05 nm incident radiation and a 50× aperture.

Electrochemical measurements. The electrochemical performances were measured by symmetrical two-electrode supercapacitor devices (CR2032 coin cells). The electrodes of YP80/G and YP80/G-P were prepared by mixing 90 wt% active materials with 10 wt% polytetrafluoroethylene (PTFE, 60 wt% aqueous solution) into a paste using an agate mortar and pestle, while YP80 electrodes were prepared by mixing active materials with PTFE and carbon black with a weight ratio of 8:1:1. After the paste was rolled into a film, it was punched into 1 cm diameter electrodes and compressed onto carbon-coated aluminum foil (current collector, 20 µm and 18 µm thick before and after compressing) under 10 MPa. After dried at 70°C for 12 h in a vacuum oven, two symmetric electrodes isolated by the separator were assembled in a stainless steel battery case with electrolyte. Celgard 3501 separators were used for aqueous and ionic electrolyte, while polypropylene separators were used for organic electrolyte. The assembly of cells with $EMIMBF_4$ or $TEABF_4$/AN electrolyte were done in a glove box filled with Ar. All the cells were tested at room temperature. Electrochemical impedance spectroscopy (EIS), cyclic voltammetry (CV) and galvanostatic charge-discharge measurements were conducted on the electrochemistry workstations (Metrohm, Switzerland). Cyclic stability was

tested at a current density of 2 A g^{-1} using LAND (Wuhan, China). The gravimetric capacitances were calculated from charge-discharge curves using the Equation (1)

$$C_g\,(\mathrm{F\ g^{-1}}) = 2I\Delta t/m\Delta U \tag{1}$$

where I is the constant current applied, A; Δt is the discharge time, s; ΔU is the operating voltage, V; and m is the net mass of the active material of one electrode, g.

The volumetric capacitances were calculated as:

$$C_v\,(\mathrm{F\ cm^{-3}}) = \rho \times C_g \tag{2}$$

where C_g is the specific capacitance of the electrode, F g^{-1}; and ρ is the density of the dried calendared electrode measured according to the formula $\rho = m/Sd$, where S (cm^2) and d (cm) are the area and thickness of the calendared electrode, respectively.

The gravimetric and volumetric energy density were calculated as:

$$E_g\,(\mathrm{Wh\ kg^{-1}}) = C_g\,(\Delta U)^2 / (2 \times 4 \times 3.6) \tag{3}$$

$$E_v\,(\mathrm{Wh\ L^{-1}}) = E_g \times \rho \tag{4}$$

where C_g is the gravimetric capacitances of the electrode, F g^{-1}; ΔU is the operating voltage, V; and ρ is the density of the electrode, g cm^{-3}.

The gravimetric and volumetric power density were calculated from the following equations:

$$P_g\,(\mathrm{Wh\ kg^{-1}}) = 3600 \times E_g/\Delta t \tag{3}$$

$$P_v\,(\mathrm{Wh\ L^{-1}}) = P_g \times \rho \tag{4}$$

where E_g is the gravimetric energy density of the electrode, Wh kg^{-1}; Δt is the discharge time, s; and ρ is the density of the electrode, g cm^{-3}.

II. Results

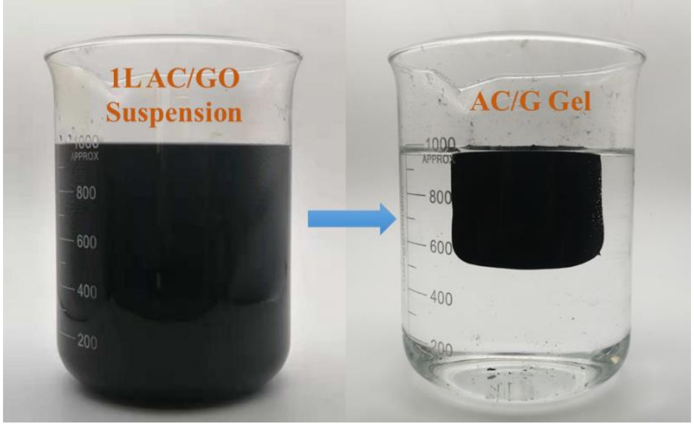

Figure S1. Scalability of this densification method. Photographs of a 1L monolithic wet gel, prepared from 1L suspension containing 1 g AC and 1 g GO.

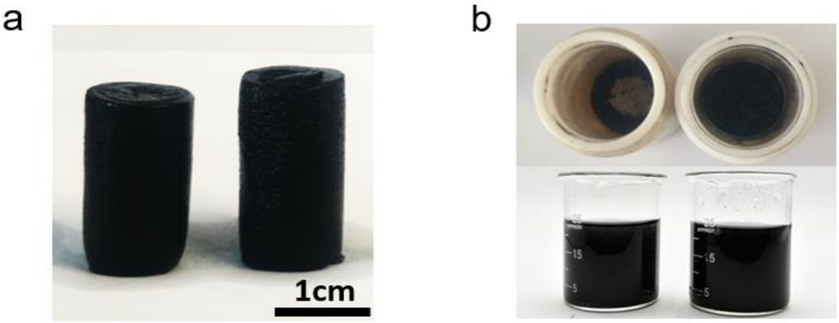

Figure S2. Preparation of AC/G by hydrothermal method. (a) Photograph of AC/G gels obtained from hydrothermal reduction at 180℃ for 6 h in 50m water/ethanol, the raw material ratios of AC to GO were 5:5 and 7:3, respectively. (b) Photographs of the AC precipitate at the bottom of the autoclaves and redispersion of precipitate in ethanol.

Table S1. Preparation details of YP80/G with different mass ratio

	**Raw material ratios YP80 : GO**	**YP80 loss**	**YP80 : YP80/G**
Low-temperature assembly	0:10	0	0
	4:6	0	67.5%
	5:5	0	76.5%
	7:3	0	88.2%
	8:2	0	93.8%
	9:1	0	96.7%
Hydrothermal assembly	5:5	46%	56.1%
	7:3	45%	75.9%

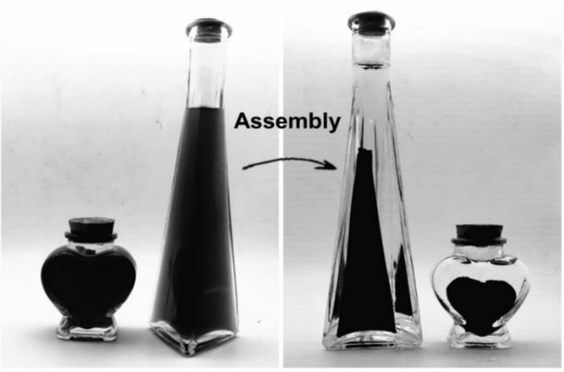

Figure S3. Heart- and tower-shaped hydrogel formed in corresponding containers. The G sheets constructed a 3D network in the container and completed this gelation process before inward shrinkage, so the shapes of hydrogel were same with the containers.

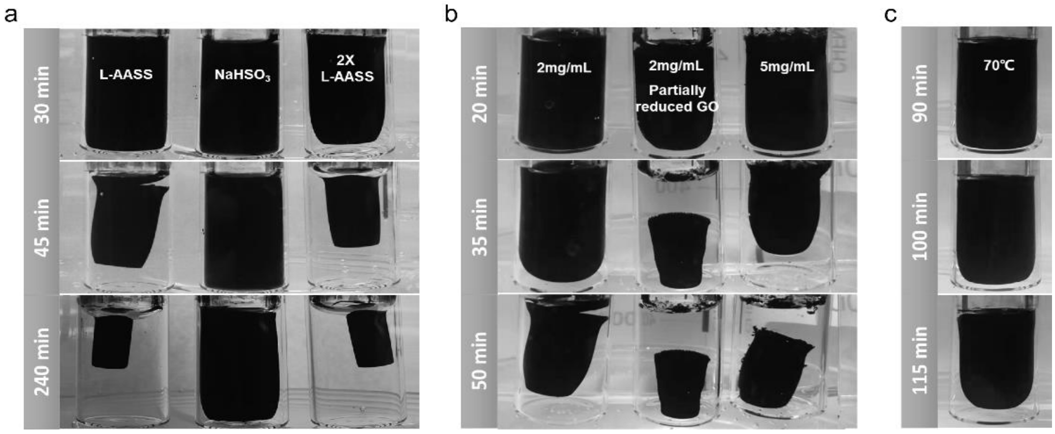

Figure S4. Gelation speed can be adjusted by (a) reductant, (b) initial oxygen content and concentration of GO and (c) temperature. Different reductants including 0.01M L-AASS, 0.01M sodium hydrogen sulfite (NaHSO3) and 0.02M L-AASS were used.

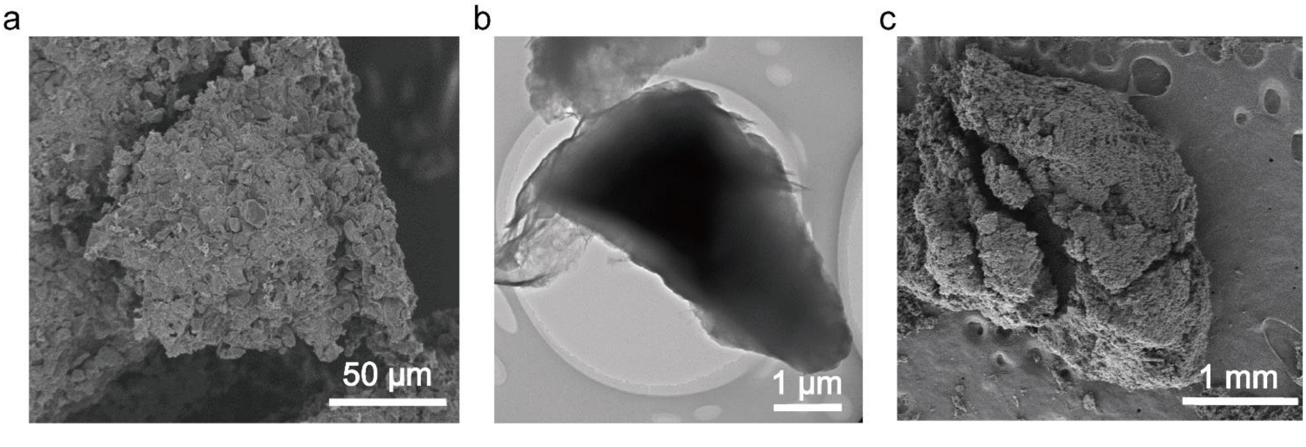

Figure S5. Micromorphology of AC/G and AC/G foam dried by freeze drying. (a) SEM and (b) TEM images of AC/G. AC particles are packed into a dense monolith as pomegranate bulk whose packing structure is free of voids. AC particles are tightly wrapped by graphene sheets. (c) SEM characterization of AC/G foam. The Low-magnification SEM image demonstrates the loose foam structure of the composite.

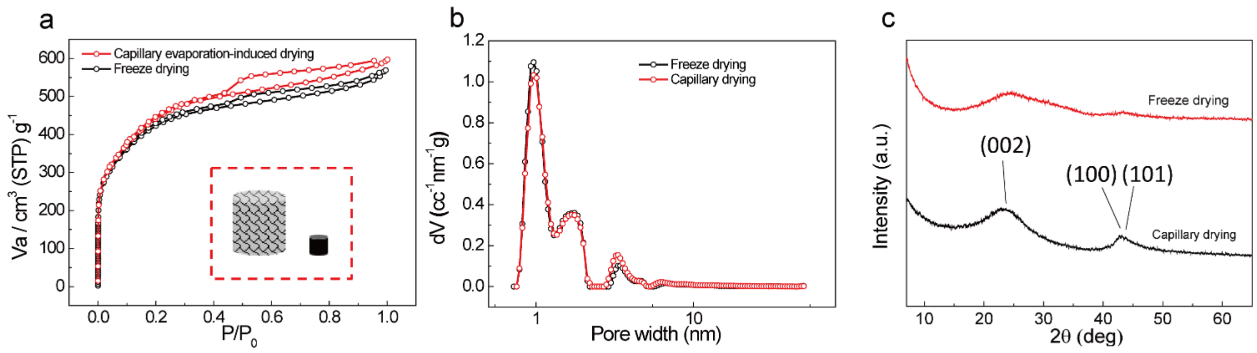

Figure S6. Comparison of AC/G composites dried by different methods. (a) N_2 adsorption-desorption isotherms of AC/G dried by capillary evaporation-induced drying and freeze drying. (b) Pore size distributions (DFT). (c) XRD patterns.

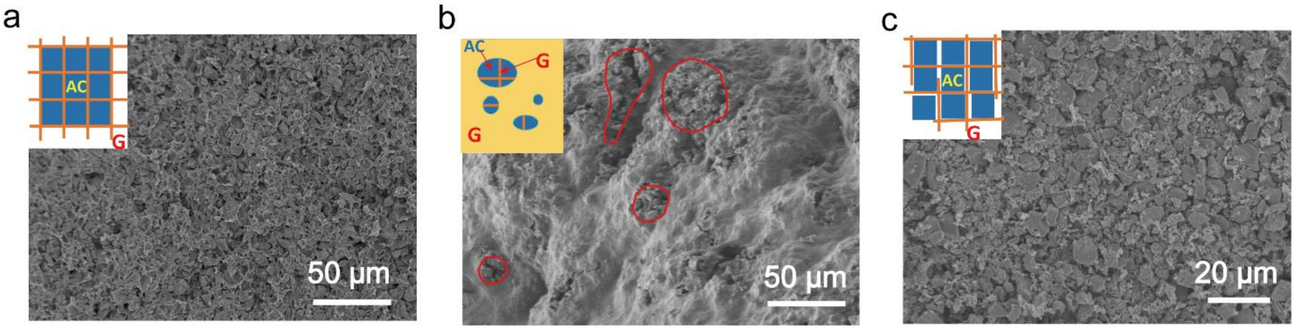

Figure S7. SEM images of AC/G with and without adequate G. Compact AC/G with well-connected and highly compressing graphene network seen from (a) vertical and (b) horizontal orientation of graphene nanosheets, the red circles mark the exposed AC and the brighter areas outside these circles are outspread graphene nanosheets. (c) Composite structure of AC/G without adequate graphene to form successive and integral 3D network.

Table S2. Pore parameters of different samples.

	**G**	**67.5% YP80/G**	**76.5% YP80/G**	**88.2 % YP80/G**	**93.8% YP80/G**	**YP80**
BET surface area (m^2 g^{-1})	521	1503	1655	1902	2011	2165
Total pore volume (cm^3 g^{-1})	0.33	0.54	0.58	0.66	0.70	0.72
Dominated pore size	~1 nm micropores, 3~4 nm mesopores	~1 nm and 1.3~2 nm micropores, 3~4 nm mesopores				

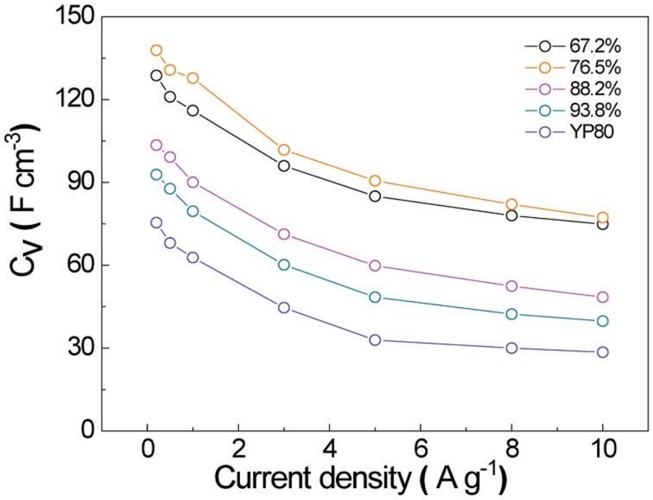

Figure S8. Volumetric capacitances of the composite electrodes with different YP80 mass loadings in $EMIMBF_4$.

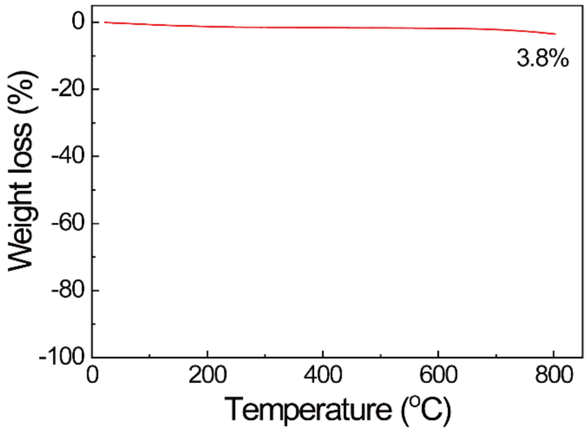

Figure S9. TGA curve of YP80. A 3.8% accumulated weight loss indicates a tiny amount of oxygen functional groups in YP80.

Table S3. Compositions of YP80 and YP80/G obtained by XPS.

	C (at.%)	O (at.%)
YP80	96.15	3.85
YP80/G	95.28	4.72

Table S4. Pore parameters of uncompressed and compressed YP80 powder and YP80/G electrode.

	**YP80 Powder**	**YP80 Powder-10**	**YP80/G Electrode**	**YP80/G Electrode-10**
BET surface area (m^2 g^{-1})	2165	2147	1492	1435
Total pore volume (cm^3 g^{-1})	0.72	0.70	0.53	0.52
Dominated pore size	~1 nm and 1.3~2 nm micropores, 3~4 nm mesopores		1~2 nm micropores, 2~3.5 nm mesopores	

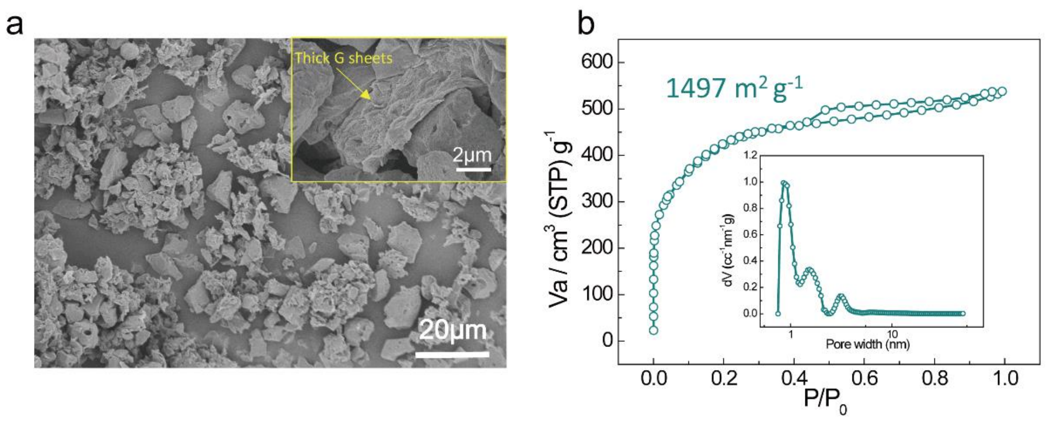

Figure S10. Characteration of YP80/G-P with 76.5% YP80. (a) SEM images of AC particles and graphene sheets, no 3D network was constructed and re-stacked graphene layers unevenly distributed among particles; (b) N_2 adsorption-desorption isotherm, inset: pore size distributions (DFT), the surface area is less than that of YP80/G due to restacking of graphene sheets.

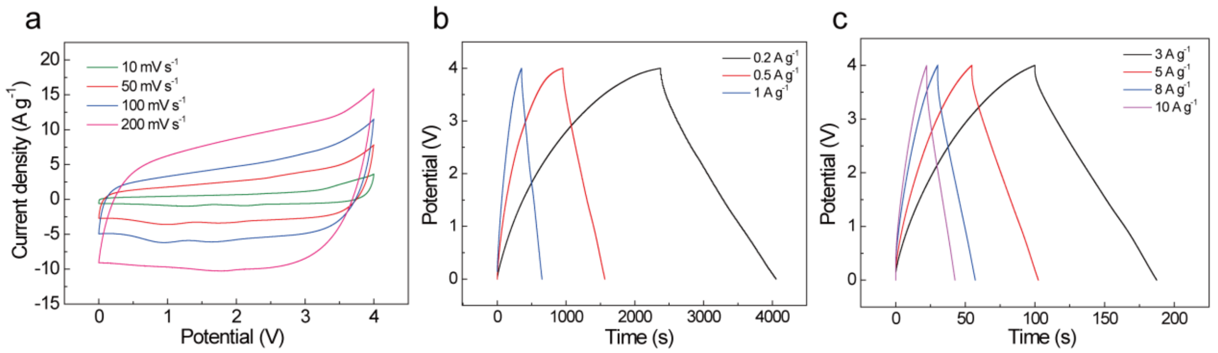

Figure S11. Electrochemical performance of the optimized YP80/G in $EMIMBF_4$. (a) Cyclic voltammograms at different scan rates; Charge-discharge curves at current densities of (b) 0.2-1 A g^{-1} and (c) 3-10 A g^{-1}.

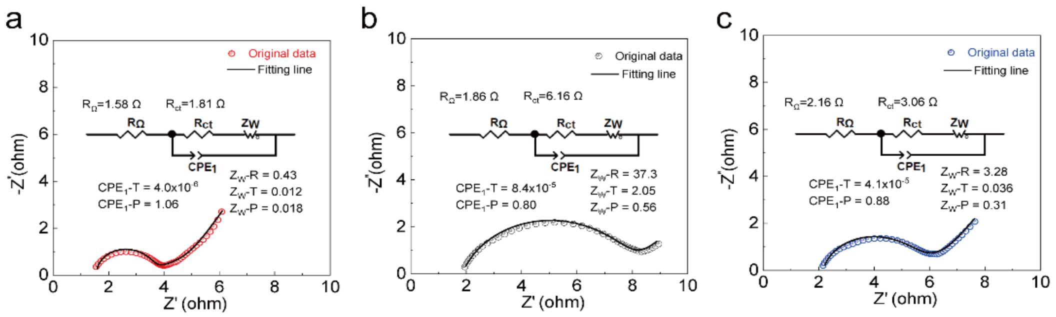

Figure S12. The original EIS plots and fitting lines of YP80/G (a), YP80 (b) and YP80/G-P (c).

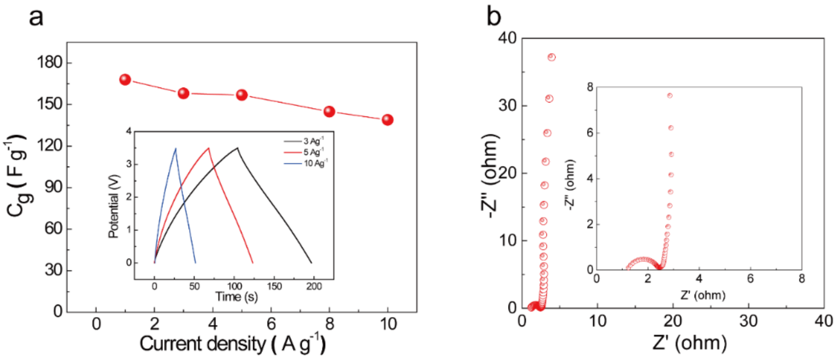

Figure S13. Electrochemical performance of the optimized YP80/G electrode in 1M $EMIMBF_4$/AN. (a) Gravimetric capacitances at the current densities from 1 to 10 A g^{-1}, inset: charge-discharge curves at current densities of 3-10 A g^{-1} ; (b)) Nyquist plot, inset: the close-up view of the high-frequency regime.

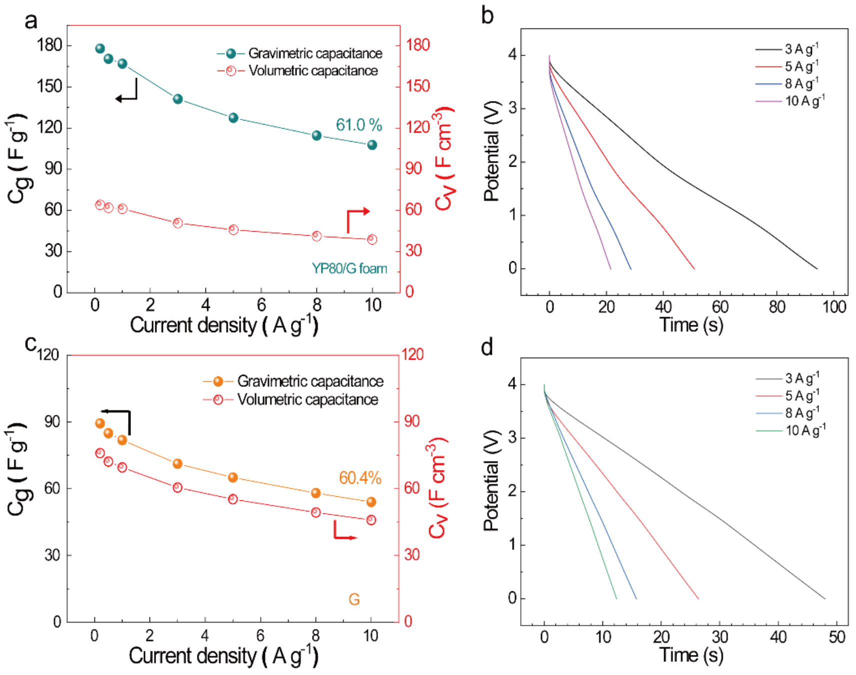

Figure S14. Electrochemical performances of 76.5% YP80/G composite obtained by freeze-drying (YP80/G foam) and G. Gravimetric and volumetric capacitances of YP80/G foam (a) and G (c) at the current densities from 0.2 to 10 A g^{-1}; Discharge curves of YP80/G (b) and G (d) at different current densities.

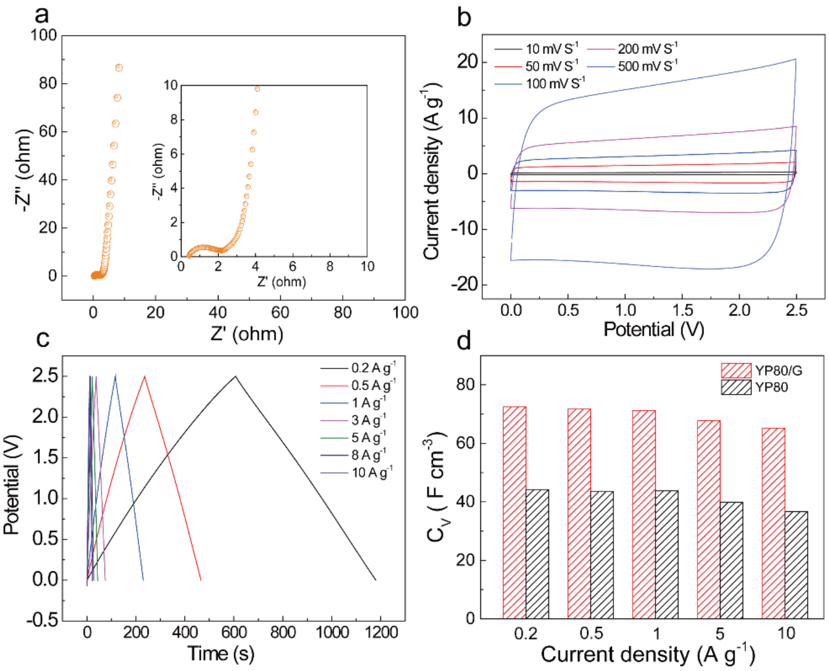

Figure S15. Electrochemical performance of the optimized YP80/G electrode in organic electrolyte. (a) Nyquist plot of YP80/G in $TEABF_4$/AN, inset: the close-up view of the high-frequency regime. (b) CV curves at voltage scan rates of 10-500 mV S^{-1}, rectangular CV shapes observed at all rates indicating more efficient double-layer formation compared with that in ionic electrolyte. (c) Charge-discharge curves at current densities of 0.2-10 A g^{-1}. (d) Volumetric capacitances of YP80/G and YP80 versus different current densities.

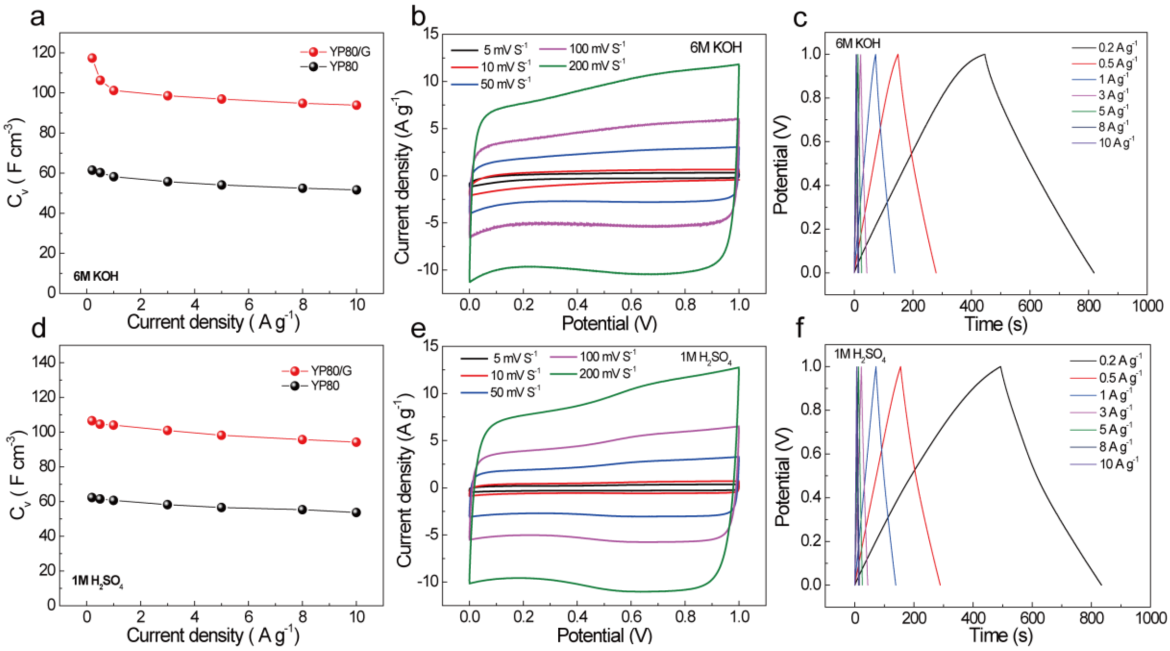

Figure S16. Electrochemical performance of the optimized YP80/G without heat-treatment in aqueous electrolyte. Volumetric capacitances of YP80/G and YP80 versus different current densities in (a) KOH and (d) H_2SO_4, YP80/G shows much higher volumetric capacitances both in acid and basic electrolyte. CV and charge-discharge curves of YP80/G in (b, c) KOH and (e, f) H_2SO_4.

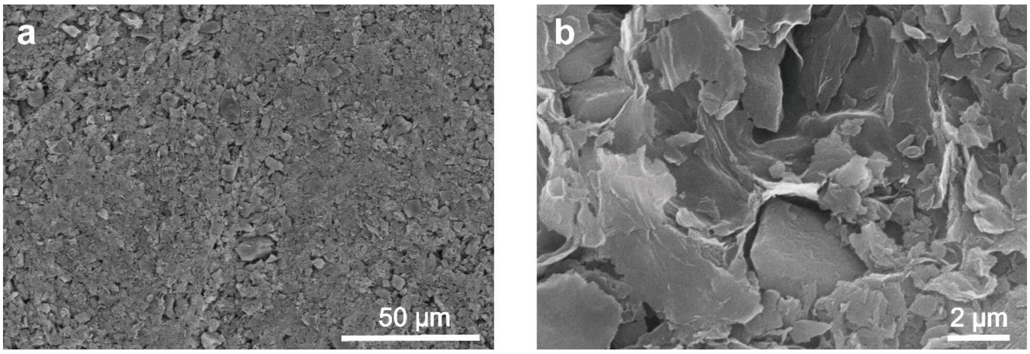

Figure S17. (a, b) SEM images of YP80/G electrode after cycling, indicating that the dense packing structure was well retained after the electrochemical test.

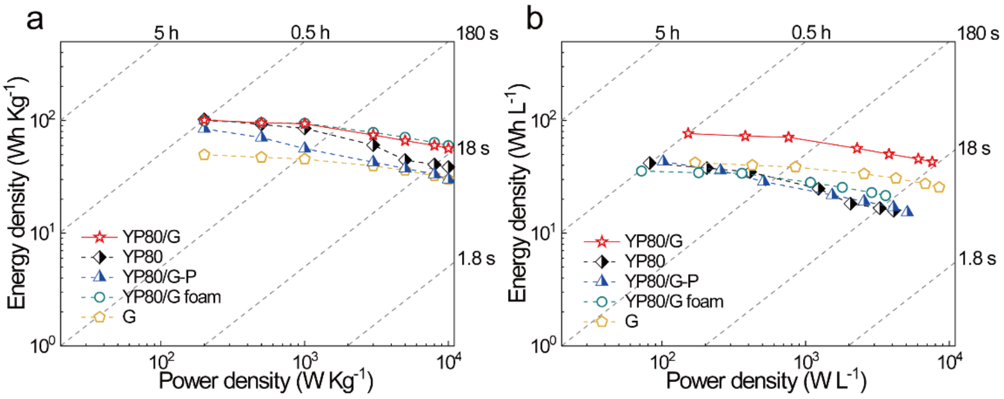

Figure S18. Gravimetric (a) and volumetric (b) Ragone plots of YP80/G, YP80, YP80/G-P, YP80/G foam and G.

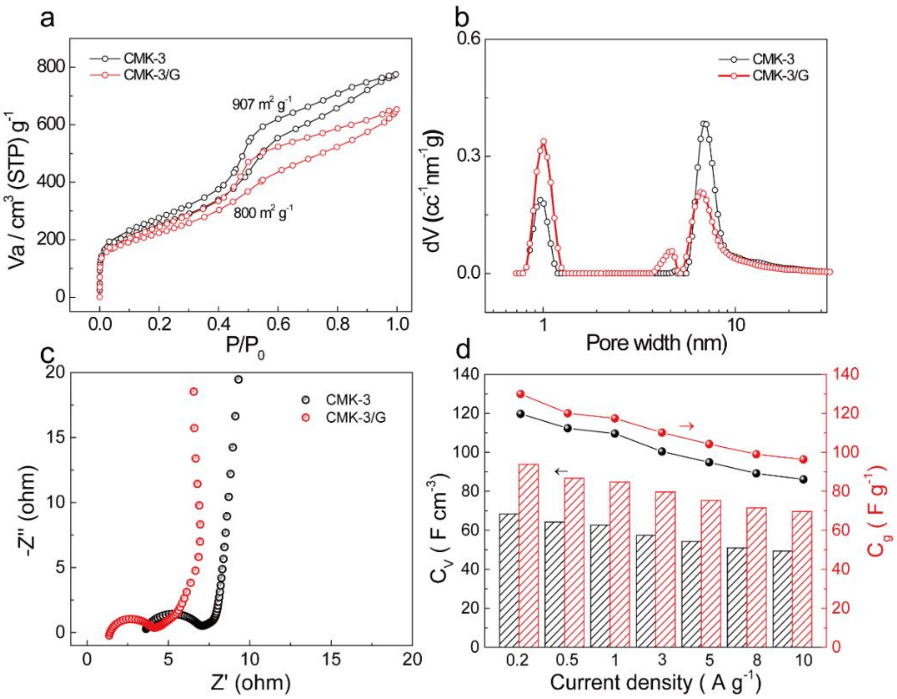

Figure S19. CMK-3/G Vs CMK-3 in $EMIMBF_4$. (a) N_2 adsorption-desorption isotherms, BET SSAs of pure CMK-3 and CMK-3/G with 76.5% CMK-3 are 907 m^2 g^{-1} and 800 m^2 g^{-1}, respectively. (b) Pore size distributions (DFT). (c) Nyquist plots. (d) Volumetric and gravimetric capacitances versus different current densities.

Table S5. Performance of reported carbon-based supercapacitors.

Materials	Electrode density (g cm^{-3})	C_q (F g^{-1})	C_v (cm g^{-3})	Electrolyte, voltage	Current density (A g^{-1})	E_q (Wh kg^{-1})	E_v (W L^{-1})	Ref.
Commercial activated carbon	~0.5-0.7	80~120	48~84	NA , 2.5~2.8 V	NA	20~30	12~21	1
AC/Mxene	0.306	126	38	Et_4NBF_4/AN, 2.0V	0.1	17.5	5.4	2
Holey graphene film	1.2	45	54	EMIMTFSI, 2.5 V	3	9.8	11.8	3
Activated graphene	0.4	231	92	$EMIMBF_4$, 3.5 V	1	98	39.2	4
Curved graphene	0.3	154	46.1	$EMIMBF_4$, 4V	1	85.6	25.7	5
Oriented graphene hydrogel	0.069	271	19	$EMIMBF_4$, 4V	0.1	151.7	10.5	6
Laser-scribed graphene	0.048	276	13.2	$EMIMBF_4$, 4V	5	117.4	5.6	7
Carbide derived carbon	0.53	160	85	EMIMTFSI, 3V	0.3	50	26.5	8
a-MEGO	0.36	165	59.8	$BMIMBF_4$/AN, 3.5V	1.4	70.6	25.4	9
Compressed a-MEGO	0.75	147	110	$BMIMBF_4$/AN, 3.5V	1.2	63	48	10
as-MEGO	0.59	173	102	EMIMTFSI/AN, 3V	2.1	74	44	11
CNTs arrays	0.5	160	80	Et_4NBF_4/PC, 4V	1	94	47	12
Chemically linked r[GO/CNT]	1.5	110	165	TEABF4, 3 V	0.5	34.4	51.6	13
YP80/G	0.76	181	138	$EMIMBF_4$, 4V	0.2	101	77	This work
		168	128	$EMIMBF_4$, 4V	1	93	71	
		102	77	$EMIMBF_4$/AN, 4V	10	57	43	
		139	105	$EMIMBF_4$/AN, 3.5V	10	59	45	

References

[1] Y. X. Xu, K. X. Sheng, C. Li, G. Q. Shi, *ACS Nano* **2010**, 4, 4324.

[2] L. Yu, L. Hu, B. Anasori, Y.-T. Liu, Q. Zhu, P. Zhang, Y. Gogotsi, B. Xu, *ACS Energy Lett.* **2018**, 3, 1597.

[3] X. Han, M. R. Funk, F. Shen, Y.-C. Chen, Y. Li, C. J. Campbell, J. Dai, X. Yang, J.-W. Kim, Y. Liao, J. W. Connell, V. Barone, Z. Chen, Y. Lin, L. Hu, *ACS Nano* **2014**, 8, 8255.

[4] L. Zhang, F. Zhang, X. Yang, G. Long, Y. Wu, T. Zhang, K. Leng, Y. Huang, Y. Ma, A. Yu, Y. Chen, *Sci. Rep.* **2013**, 3, 1408.

[5] C. Liu, Z. Yu, D. Neff, A. Zhamu, B. Z. Jang, *Nano Lett.* **2010**, 10, 4863.

[6] X. Yang, J. Zhu, L. Qiu, D. Li, *Adv. Mater.* **2011**, 23, 2833.

[7] M. F. El-Kady, V. Strong, S. Dubin, R. B. Kaner, *Science* **2012**, 335, 1326.

[8] C. Largeot, C. Portet, J. Chmiola, P.-L. Taberna, Y. Gogotsi, P. Simon, *J. Am. Chem. Soc.* **2008**, 130, 2730.

[9] Y. Zhu, S. Murali, M. D. Stoller, K. J. Ganesh, W. Cai, P. J. Ferreira, A. Pirkle, R. M. Wallace, K. A. Cychosz, M. Thommes, D. Su, E. A. Stach, R. S. Ruoff, *Science* **2011**, 332, 1537.

[10] S. Murali, N. Quarles, L. L. Zhang, J. R. Potts, Z. Tan, Y. Lu, Y. Zhu, R. S. Ruoff, *Nano Energy* **2013**, 2, 764.

[11] T. Kim, G. Jung, S. Yoo, K. S. Suh, R. S. Ruoff, *ACS Nano* **2013**, 7, 6899.

[12] A. Izadi-Najafabadi, S. Yasuda, K. Kobashi, T. Yamada, D. N. Futaba, H. Hatori, M. Yumura, S. Iijima, K. Hata, *Adv. Mater.* **2010**, 22, E235.

[13] N. Jung, S. Kwon, D. Lee, D. M. Yoon, Y. M. Park, A. Benayad, J. Y. Choi, J. S. Park, *Adv. Mater.* **2013**, 25, 6854.
